# The Frequency, Magnitude, and Spatial Distribution of Heart Rot in Dominant Temperate Tree Species in a Forest Dynamics Plot

**DOI:** 10.1002/ece3.71329

**Published:** 2025-04-17

**Authors:** Hunter Gonzalez, Ally O'Neill, Michael Parent, Debit Datta, Nathan G. Swenson

**Affiliations:** ^1^ University of Notre Dame Environmental Research Center, University of Notre Dame Indiana USA; ^2^ Department of Biological Sciences University of Notre Dame Indiana USA

## Abstract

The composition, dynamics, and health of forest tree communities are governed by interactions with the abiotic and biotic environment. Fungi are critical biotic interactors that play an increasingly appreciated role in forest tree health, particularly with respect to mycorrhizal and pathogenic fungi. Heart rot fungi, while known to infect a large fraction of the individuals in managed stands, have been considerably understudied in tree community ecology. Heart rot has been predicted to form hotspots in the forest due to crown or bole damage and/or soil moisture gradients and is expected to vary across species due to life‐history differences. To address this knowledge gap, we quantified the incidence, magnitude, and spatial distribution of heart rot in 328 individual trees with diameters greater than or equal to 10 cm across the six most dominant tree species in a mixed broadleaf temperate forest dynamics plot. The results show that 71% of individuals display some degree of heart rot in this natural community. The incidence of heart rot did not significantly vary across species despite their life history strategy differences, but one species had a significantly higher magnitude of heart rot in infected individuals. Lastly, heart rot was spatially clustered across species, but heart rot incidence and magnitude were not related to soil moisture, indicating the importance of crown and bole breakage likely promoted by severe weather. The present study has conducted the first spatially explicit study of heart rot incidence and magnitude in a natural forest tree community. We demonstrate that over two‐thirds of the large trees in the forest studied have some degree of heart rot indicating their widespread, but underappreciated, incidence in tree communities. We demonstrate that heart rot is nonrandomly distributed in this community and that spatial clustering of heart rot in forests is most likely due to hotspots of individual tree damage and not gradients in soil moisture.

## Graphical abstract

The present study conducted the first spatially explicit study of heart rot incidence and magnitude in a natural forest tree community. We demonstrate that over two thirds of every large tree in the forest studied have some degree of heart rot, indicating their widespread, but underappreciated, role in tree communities. We demonstrate that heart rot is nonrandomly distributed in this community and that spatial clustering of heart rot in forests is most likely due to hotspots of individual tree damage and not gradients in soil moisture.

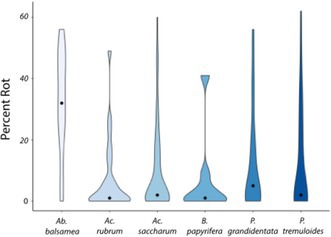

## 1 Introduction

Individual‐level tree growth and mortality underlie forest dynamics, productivity, and health (Yang et al. 2018; Swenson et al. 2020). The abiotic and biotic drivers of tree mortality have drawn considerable interest. Recent work clearly elucidates the important impacts of water limitation or drought on individual tree distributions and performance and forest health (e.g., Anderegg et al. 2013; Swenson et al. 2017; Swenson et al. 2023; Choat et al. 2018). Similarly, declines in tree performance are often linked to higher local conspecific density (e.g., Johnson et al. 2012; Comita et al. 2014). Increasingly, fungi have been suggested as the factor causing these density‐dependent declines in performance (e.g., Bagchi et al. 2010; Chen et al. 2019; Milici et al. 2024) and there are a handful of key studies that draw mechanistic linkages (e.g., Mangan et al. 2010; Bennett et al. 2017). Thus, our understanding of tree community structure and dynamics requires an increasing focus on fungal interactions and how they may relate to key abiotic resource gradients through space and time.

A key group of fungi that are understudied in tree community ecology are the heart rot fungi (Wagener and Davidson 1954). Heart rot fungi infect trees via vegetative growth of mycelium through root contact or grafts with neighboring infected trees or via spores through wounds. These fungi decay the heartwood, weakening the stem, leading to mechanical failure and may also lead to declines in growth rates and/or reproduction (Hepting and Fowler 1962; Mook 1969). For example, Datta et al. (2025) have shown that the presence of tree cavities in a tropical rainforest significantly impacted individual tree growth rates. The loss of merchantable lumber due to heart rot is well known to silviculturists, and the impacts of the incidence of heart rot on estimates of above ground biomass have been studied by forest ecologists (e.g., Basham 1991), but the importance of heart rot fungi for tree community structure and dynamics in natural stands is understudied (Gilbert et al. 2016).

An entry point into understanding the importance of heart rot fungi on tree community structure and dynamics is quantifying the incidence of heart rot across species and the spatial distribution of heart rot in natural stands along key resource axes. For example, there is some evidence that heart rot may be less common in wetter and lower elevations due to anoxic conditions in the soil (Basham 1967; Basham 1973; Basham 1991) and additional evidence that soil type may be as or more important (Garbelotto and Gonthier 2013). Such a relationship may cause spatial clustering in heart rot rates in the forest. Spatial clustering in heart rot across individual trees in a natural stand may also be caused by what is termed by Hennon (1995) as the “heart rot‐bole breakage‐wound cycle”. Under this scenario, a tree that is infected with heart rot fungi falls due to the lack of its core structural stability and strikes and wounds neighboring healthy trees. The wounded and previously healthy trees are then infected by spores of heart rot fungi and heart rot then occurs in those trees (Hennon 1995). This mechanism should cause spatial clusters or hotspots of heart rot that may be correlated with underlying abiotic gradients across species. Species, themselves, may also be expected to have varying incidence and magnitude of heart rot due to their life history differences. For example, fast growing species with acquisitive functional strategies may be more susceptible to pests and pathogens, while slower growing species may be better defended and less susceptible to pests and pathogens. Furthermore, fast growing species are more likely to be found in areas with canopy damage (i.e., areas where trees are more likely to have wounds serving as conduits for fungal spore colonization).

A barrier to studying heart rot in forest ecology has been the development of efficient and relatively nondestructive methods for quantifying heart rot. Some of the best studies of heart rot rates in stands have resulted from the harvesting of entire trees or even entire stands (e.g., Basham 1991; Heineman et al. 2015). This is not a feasible approach in most ecological study sites. An alternative to whole tree harvesting is to utilize an increment borer, but this approach is labor‐intensive and fairly invasive (Heineman et al. 2015; Yang et al. 2015).

Recent advances in sonic tomography have removed many of the above barriers to studying heart rot in natural stands (Gilbert et al. 2016). Specifically, sonic tomography is a less‐invasive method that provides two‐ or three‐dimensional scans of stems. This is accomplished by attaching sensing probes to the periphery of the stem. The probes are struck by a hammer to send sonic waves through the stem to the other probes. Sequential striking of probes and deviations from the expected speed at which sonic waves should reach the other probes allow for the mapping of intact and decaying wood. Thus, sonic tomography offers a minimally invasive and scalable approach for quantifying the incidence and magnitude of heart rot across individual trees in a natural stand.

This study leverages sonic tomography to provide fundamental information regarding the distribution of heart rot incidence and magnitude across species and space in a large temperate forest dynamics plot. The specific questions we ask are: (i) Does the incidence and magnitude of heart rot vary across dominant tree species in the forest plot?; (ii) are there hotspots of spatial clustering in heart rot within and across dominant tree species in the forest plot?; and (iii) is the distribution of heart rot for dominant species associated with soil moisture gradients?

## 2 Methods

### 2.1 Study Site

This study was conducted at the University of Notre Dame Environmental Research Center (UNDERC). UNDERC is a 3035‐ha property straddling the borders of the states of Wisconsin and Michigan. This temperate mixed deciduous forest borders the Ottawa National Forest and has experienced few major disturbances since the property was donated to the University in the 1930's. A low level of disturbance in the forest is important to heart rot studies as heart rot is a slow, persistent process, and occurs at a higher frequency in areas that have experienced human disturbance (Hennon 1995). The individual trees studied are located within UNDERC's forest dynamics plot (FDP), which is an area of 400 m × 400 m. The FDP is staked every 20 m, was initiated in the summer of 2022, and has had all trees with a diameter at breast height (DBH) greater than or equal to one cm measured, tagged, and identified to species.

A total of 400 20 m × 20 m subplots exist within the UNDERC FDP. We randomly selected 15 of these subplots for this study. Inside each of these 15 plots, we investigated all trees with a DBH of at least 10 cm. There were a total of 330 trees from seven species in the 15 subplots that met this diameter cutoff. One species, *Prunus serotina* , had only two individuals sampled and was, therefore, not included in this study. The six remaining species were: *Abies balsamea* (Pinaceae), *Acer rubrum* (Sapindaceae), *Ac. saccharum*, *Betula papyrifera* (Betulaceae), *Populus grandidentata* (Salicaceae), and *P. tremuloides* . These six species are the most abundant trees in the UNDERC FDP by abundance and basal area.

The 328 trees studied were spatially mapped to facilitate downstream spatial analyses. First, the UNDERC FDP was professionally surveyed and staked at every 20 m. Next, we used one 50 m tape to locate the position of each tree within a subplot on the southern border of the subplot (i.e., a position on the *x*‐axis). Then, a second tape and a compass were used to run a line directly north to the stem of the target tree to provide the y‐axis coordinate. These coordinates, combined with the UTM coordinates of the professionally surveyed stakes, allowed us to map the geospatial location of all 328 trees in the study.

### 2.2 Soil Moisture Inference

A soil moisture map for the UNDERC FDP was inferred by kriging field‐based soil core samples with derived values of elevation. Soil cores were collected using an 8‐inch soil corer every 40 m within the UNDERC FDP during the summer of 2024. Cores were taken a minimum of 48 h after the most recent rainfall, with most being taken between 48 to 72 h after the last significant rainfall. Gravimetric analysis with 10 g subsamples dried at 105°C for 24 h was used to determine soil moisture. All core samples were weighed and placed in the drying oven within 6 h of collection, but most were prepared no more than 3 h after collection to minimize loss of moisture.

A digital terrain model at the spatial resolution of one square meter was derived from airborne LiDAR data collected in 2022 by the National Ecological Observatory Network Airborne Observatory Platform (NEON AOP). The NEON AOP collects LiDAR point clouds at a 1 m^2^ resolution across the entire UNDERC property including the FDP. Ground points were classified using cloth simulation filtering (Zhang et al. 2016) and the terrain was then interpolated from the ground points using a triangulation algorithm in the lidR package (Roussel et al. 2020). Soil moisture values were interpolated via cokriging at one square meter resolution, with elevation included as a covariate to define the trend model using the geoR package (Ribeiro et al. 2007). The kriging result was converted to a raster and used in the analyses of this study. Specifically, predicted soil moisture values were extracted for each individual tree in the study using their geospatial position and the raster map.

### 2.3 Sonic Tomography

Sonic tomography was used to determine the percentage of heart rot in each of the 328 trees in this study. Sonic tomography uses the velocities of sonic waves running through the stem of a tree to determine the heart rot content of each tree. Due to the different densities of rotted and nonrotted wood and the presence of cavities, sound moves at different velocities through the different materials and around cavities. An ArborSonic3D Acoustic Tomograph (Agfalva, Hungry) was used to measure these soundwaves and to create two‐dimensional images of the cross‐sections of the stem of the trees which showed the tree's heart rot content. Throughout this process, the process of creating sonic tomographs was done by following the protocol described in Gilbert et al. (2016). For each eligible tree, 10 SD02 Piezo transducers were placed two centimeters into the stem of the trees at even intervals across the tree's circumference at 100 cm above the ground level. The sensors were attached to the tree following the ArborSonic 3D user manual. We note that changes in stem diameter relative to the number of transducers may introduce less precision in larger trees, but this noise is small relative to the overall signal. Then, the transducers were connected to amplifier boxes in counterclockwise order, and the amplifier boxes were connected to each other and the battery box using cables. Finally, each transducer was tapped three times with a metal hammer to create the soundwaves measured for the creation of the two‐dimensional tomograph (Gilbert et al. 2016). These data were then analyzed using the software ArborSonic 3D v. 5.3.146 to extract the percent decay of the measured stem. We used the default settings described in the ArborSonic 3D software manual with the exception that we selected the individual tree species being measured from the “Select Tree Species” drop down menu in the “Tree Properties” panel. The software was used to annotate each tree with its tag number and Latin binomial.

### 2.4 Statistical Analysis

**FIGURE 1:**
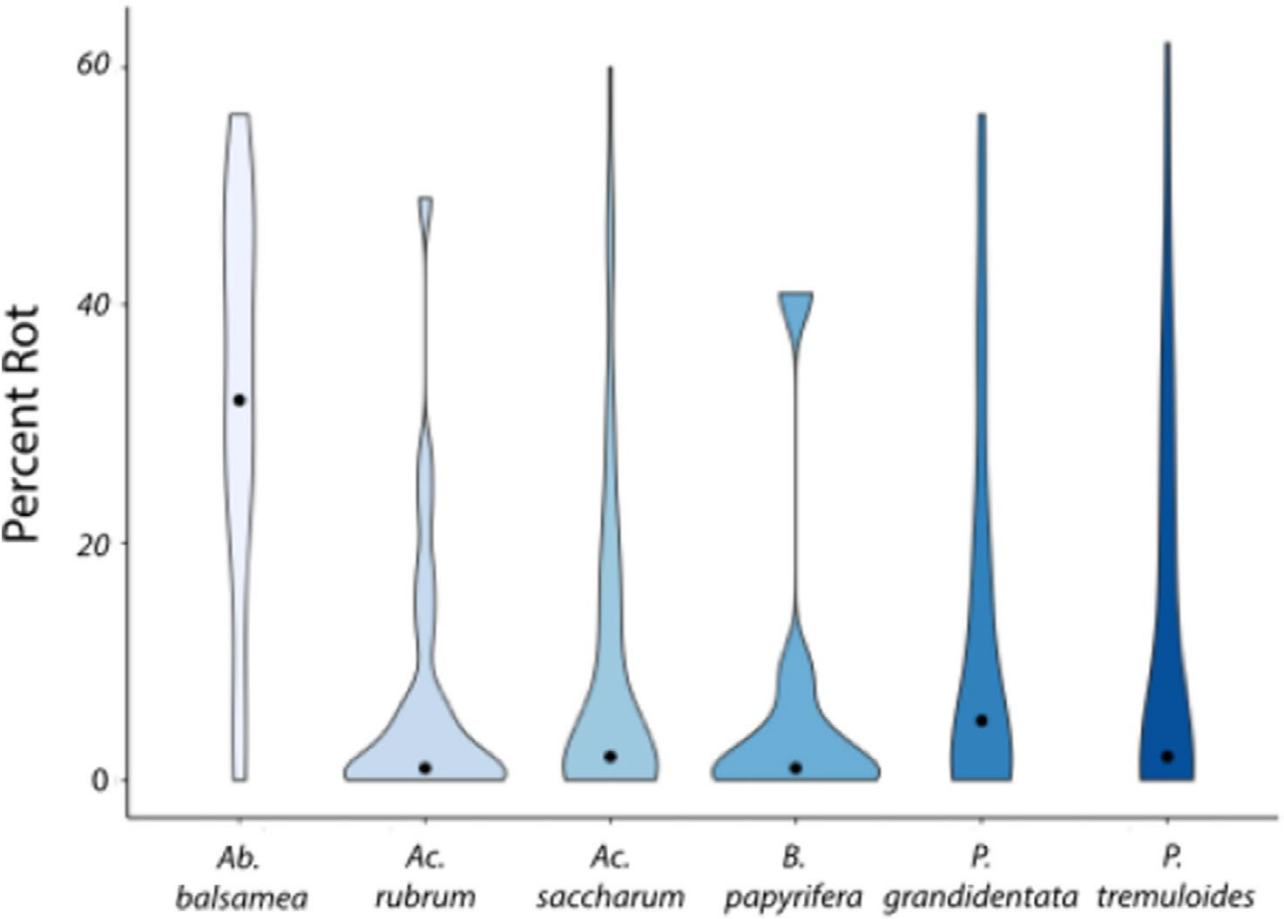
Distribution of the percentage of heart rot in stems across the six species in this study shown via violin plots.

The first question in this study was whether species differed in their incidence of heart rot. A total of 233 of the 328 (71%) trees sampled had some level of heart rot. We used a *χ* ^2^ test of equal expected frequencies to quantify whether there were species differences. Specifically, because there were unequal sample sizes per species, we set the expected frequency of the heart rot as 233 multiplied by the relative abundance of a given species in the data set (Table 1). Our second question in this study was whether species differed from one another in the average magnitude of percent heart rot in their individuals. Because the percent heart rot data were bounded and non‐normal (Figure 1) we first added 1% to each value and then logit transformed the value. These transformed values were then used in an analysis of variance followed by a Tukey Test for group differences.

**TABLE 1:** The sample sizes, incidence and percentages of heart rot across the species in this study.

Species	Number of trees sampled	Number of trees with heart rot	Mean and standard deviation of percent heart rot	Median percent heart rot
*Abies balsamea*	46	43	31.80 (17.04)	32.00
*Acer rubrum*	25	15	6.96 (11.69)	1.00
*Acer saccharum*	149	103	9.53 (13.94)	2.00
*Betula papyrifera*	8	5	7.00 (14.08)	1.00
*Populus grandifolia*	53	37	12.55 (16.33)	5.00
*Populus tremuloides*	47	30	12.28 (16.88)	2.00

Next, we wanted to know whether there was spatial clustering or autocorrelation in the incidence and magnitude of heart rot and whether the incidence and magnitude of heart rot within species was associated with soil moisture. To this end, we began by computing Mantel correlograms, which are a widely used measurement of spatial autocorrelation (Mantel 1967) performed in the R package vegan (Oksanen et al. 2013). These analyses were first computed on the incidence and log percentage of rot plus one for all individuals. Next, these analyses were repeated using only the individuals from a given species. We restricted these analyses to the only four species that had at least 30 individuals (i.e., *Ab. balsamea*, *Ac. saccharum*, *P. grandidentata* and *P. tremuloides* ). In each test, we used a Holm *p* value adjustment for multiple tests (i.e., multiple spatial scales).

Finally, as it has been suggested that soil moisture may be a correlate or driver of heart rot, we quantified whether the soil moisture around individual trees was related to their presence or absence of heart rot or the percent heart rot. To do this, we used generalized linear models with binomial and gaussian error distributions, respectively, and with spatial autocorrelation, assuming a Matérn covariance between individuals given their spatial coordinates.

## 3 Results

### 3.1 Species Comparisons of the Incidence and Magnitude of Heart Rot

A *χ* ^2^ test of equal expected incidences was used to test whether the six species had clear deviations from their expected incidences. This test indicated there were no differences in the incidences of heart rot across species (*χ* ^2^ = 4.2033, df = 5, *p* = 0.5205). While *Ab. balsamea* had 42 individuals with heart rot and the expected number was ~33, the other species have very minor deviations from the expected values. Combined, this resulted in no clear deviation overall across species from the expected incidences (Table 1).

The results of the analysis of variance test indicated at least one group mean was different from another (*F*_*5,322*_ = 12.24, *p* < 0.001). A Tukey Test for multiple comparisons indicated that the mean percent rot for *Ab. balsamea* was higher than that of all other species in this study. It also showed that those other five species had mean values that were not significantly different (Figure 1). In sum, while the incidence of heart rot was not different from that expected across species, the magnitude of heart rot in the average individual was higher in *Ab. balsamea* compared with all other species.

### 3.2 Spatial Clustering of Heart Rot Across Species

**FIGURE 2:**
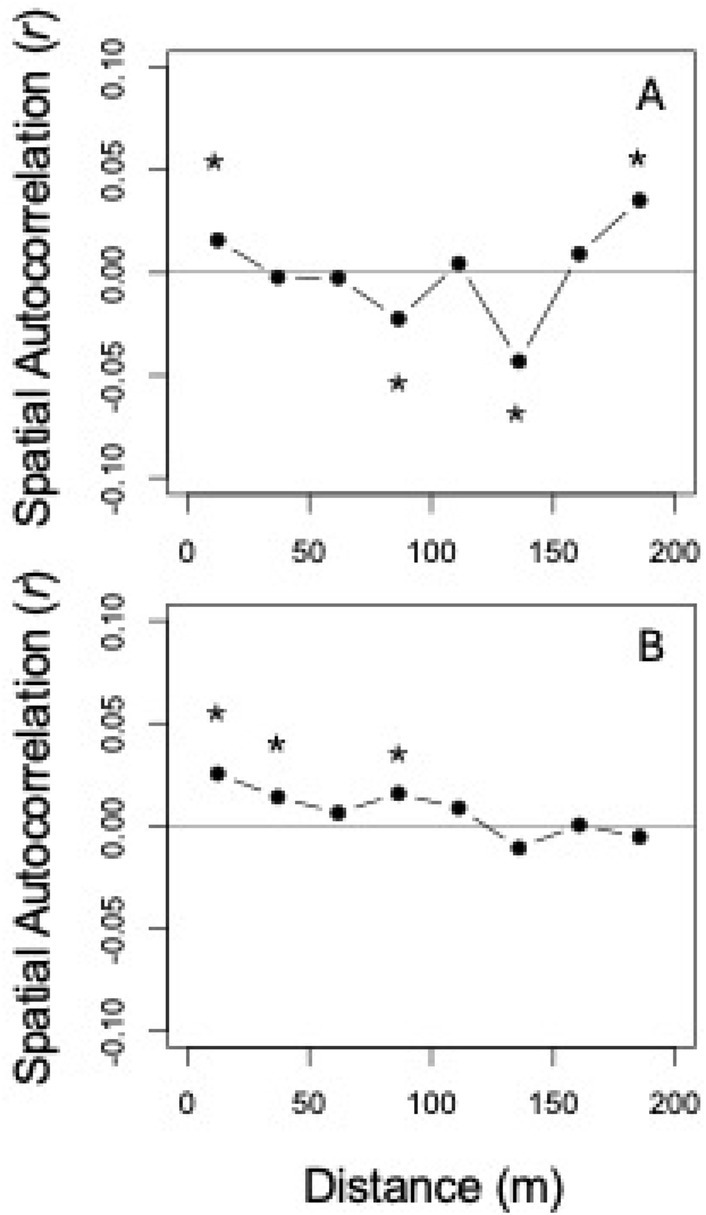
Mantel correlogram results for the incidence (A) and the percentage (B) of heart rot across all individuals in this study. The *y*‐axis reports the Mantel *r* test statistic and the *x*‐axis reports the spatial distance in meters (m). An asterisk above a point indicates that the correlation was significant (*p* < 0.05) after a Holm correction for multiple tests.

Next, we quantified the degree of spatial clustering in the incidence and percentage of heart rot for all individuals across the six different species of trees. The results for heart rot incidence demonstrated positive spatial autocorrelation at the scale of 12.47 m (i.e., roughly within the area of a 20 × 20m subplot), but this positive autocorrelation in incidence was not found again until the very largest scales (Figure 2A). There was also positive spatial autocorrelation in the percentage of heart rot within individual stems. This included the first two and the fourth spatial scales: 12.47, 37.14, and 86.53 m (Figure 2B).

### 3.3 Spatial Clustering of Heart Rot Within Species

**FIGURE 3:**
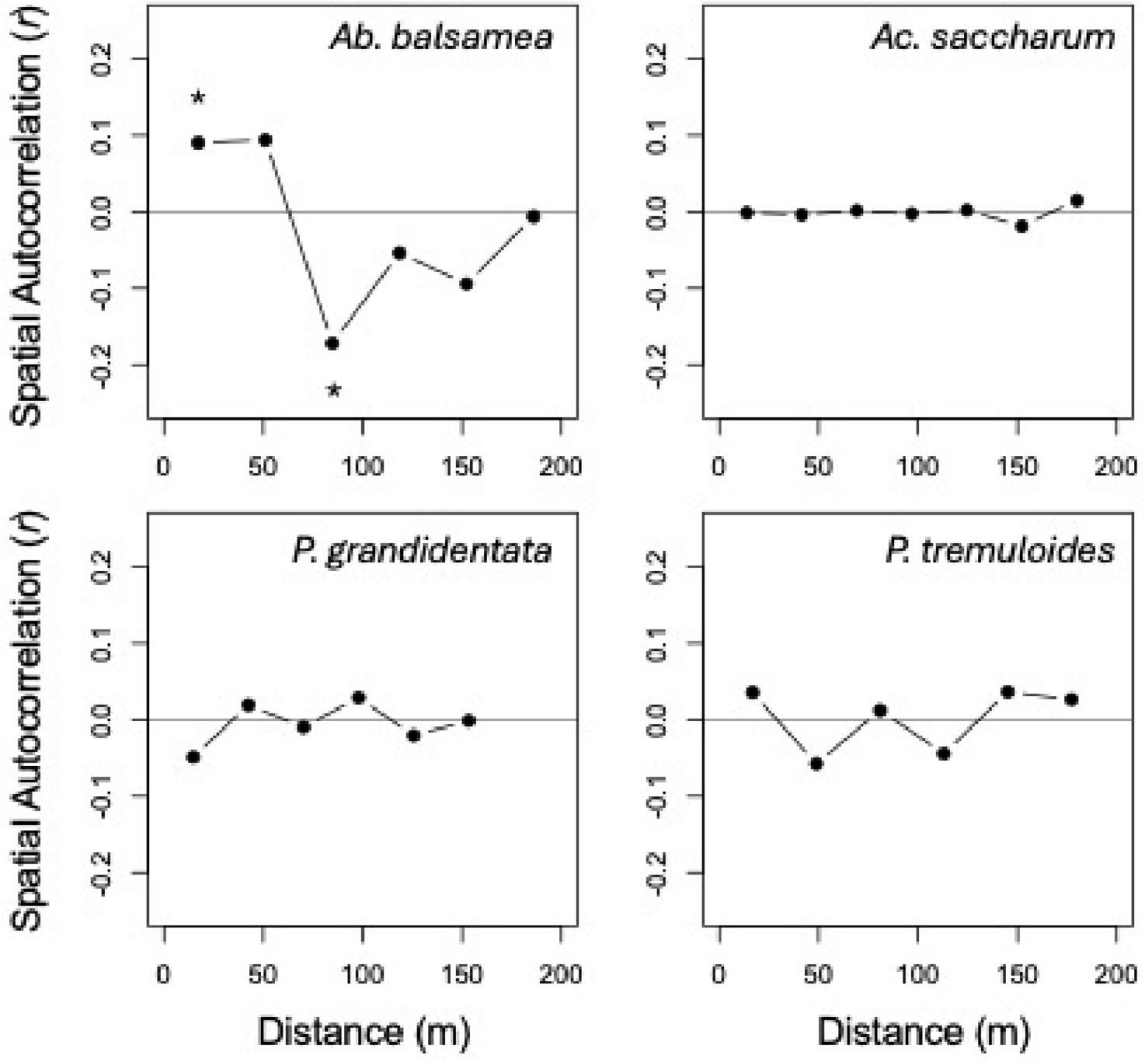
Mantel correlogram results for the incidence of heart rot for the individuals of the four species in this study with more than 30 individuals. The *y*‐axis reports the Mantel *r* test statistic, and the *x*‐axis reports the spatial distance in meters (m). An asterisk above a point indicates that the correlation was significant (*p* < 0.05) after a Holm correction for multiple tests.

**FIGURE 4:**
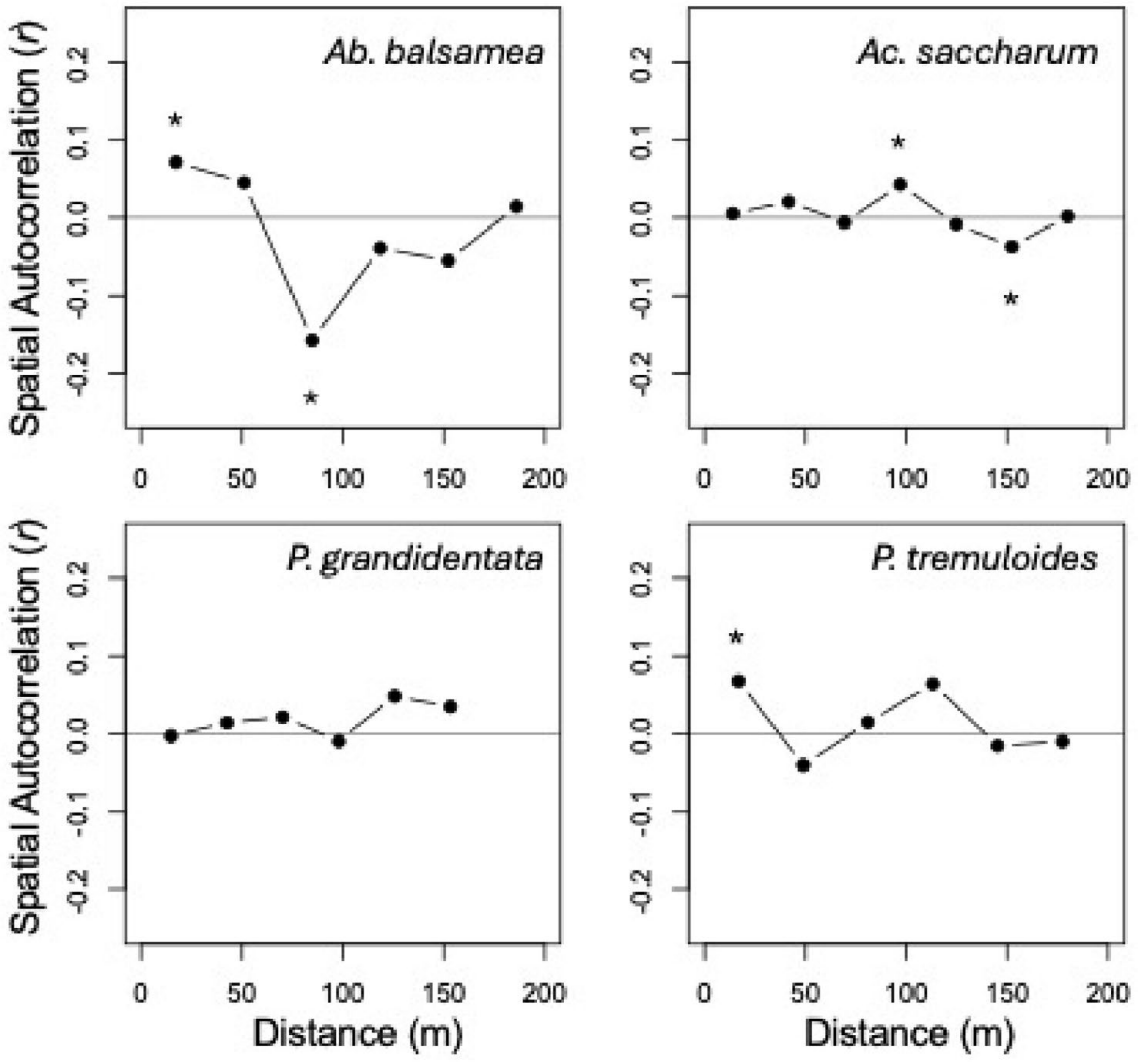
Mantel correlogram results for the percentage of heart rot for the individuals of the four species in this study with more than 30 individuals. The *y*‐axis reports the Mantel *r* test statistic and the *x*‐axis reports the spatial distance in meters (m). An asterisk above a point indicates that the correlation was significant (*p* < 0.05) after a Holm correction for multiple tests.

There were four species in our study that had greater than 30 individuals, allowing us to robustly estimate the degree of spatial clustering of heart rot within species. *Ab. balsamea* was the only species that had significant positive spatial autocorrelation in heart rot incidence (Figure 3). The scale of this autocorrelation was 17.32 m. For the percentage of heart rot within stems, there was a positive spatial autocorrelation in *Ab. balsamea* and *P. tremuloides* at the scales of 17.32 and 16.82 m, respectively. Finally, *Ac. saccharum* had a positive spatial autocorrelation in the percentage of heart rot in stems at the scale of 96.94 m (Figure 4).

### 3.4 Heart Rot and Soil Moisture

The final aspect of this study was to quantify whether the soil moisture for individual trees within a species was related to the incidence or percentage of heart rot. We found that soil moisture was not a significant predictor of the incidence or magnitude of heart rot in any of the four species with more than 30 individuals in the study.

## 4 Discussion

Fungal interactions play an increasingly appreciated role in driving tree community structure and dynamics via their impacts on individual‐level demographic outcomes (e.g., Mangan et al. 2010; Chen et al. 2019). The prevalence and economic consequences of heart rot in managed stands have been documented in the forestry literature (e.g., Basham 1991), but there are relatively few studies of heart rot in natural tree communities. Thus, we have little information about the incidence of heart rot across and within the species in communities and the potential spatial drivers of heart rot (Gilbert et al. 2016). Here, we sought to address these knowledge gaps by studying six tree species that dominate a large forest dynamics plot in a temperate mixed broadleaf forest. These species occupy nearly the entire canopy in this research site; they represent a diverse sample phylogenetically and with respect to life history strategies that are differentially distributed along key resource axes. We infer, given the results from this study, that the spatial distribution of heart rot across species in this forest is most likely driven by a heart rot‐bole breakage‐wound cycle mechanism proposed by Hennon (1995). Specifically, this mechanism produces spatial hot spots of heart rot due to infected and mechanically compromised trees falling and, in doing so, wounding neighboring trees that become infected with the fungal spores of the originally infected tree. Importantly, this process should lead to no differences in the incidence of heart rot between species because it is a purely spatial process. However, species may be expected to vary in the magnitude of heart rot in infected individuals due to interspecific life history and wood chemistry differences. In the following, we discuss the results from the three major aspects of the study.

### 4.1 Incidence and Magnitude of Heart Rot Across Species

The first goal of this study was to address whether this dominant and diverse set of species varied with respect to the incidence and magnitude of heart rot. We found that the incidence of heart rot did not vary across species. While the incidence did not vary, it is noteworthy that at least 60% of the individuals with diameters of at least 10 cm in each of the species we studied had some heart rot as detected by the sonic tomograph. This number is roughly in line with that reported in other studies (Basham 1991; Heineman et al. 2015), but it is likely far higher than what a typical tree community ecologist studying natural stands may expect. The ecological significance of such widespread heart rot and its impacts on population and community structure and dynamics in natural stands is still not well understood (Gilbert et al. 2016), but it is apparent that rot is a widespread phenomenon that demands more investigation.

The incidence of heart rot across species was consistently high in this study, but did not vary across species. The magnitude of heart rot, though, did vary across species. This result was driven by the high magnitudes of heart rot in *Abies balsamea* . Specifically, the median area of heart rot in *Ab. balsamea* was 32% whereas the other species had a median of 1%–5% (Table 1). The lack of difference in the amount of heart rot across the Angiosperm species in this study was not expected. We expected that the faster growing species (i.e., *Populus*) would likely have more heart rot due to their lighter and, potentially, less well‐defended wood as compared to more shade tolerant species (e.g., *Acer saccharum* ). It is possible that the failure to find differences among these species is due to our comparison of similarly sized individuals and not similarly aged individuals. The magnitude of heart rot increases with age (Basham 1991) and this information may allow for a useful comparison of the area of heart rot for a given age. However, we could not quantify the ages of individuals in this study as coring trees in this long‐term observational plot is not permitted and, obviously, aging trees with heart rot via cores is not feasible. The large amount of heart rot found in *Ab. balsamea* is in line with the silvicultural literature (Mook 1969). The lower medians in the other species may indicate heart rot is less important in these species, but it is noteworthy that nearly all trees in this study appeared asymptomatic and these apparently healthy trees occasionally had upward of 40%–60% of their stem decayed. The main exceptions to this were the two *Populus* species where large individuals (e.g., DBH > 30 cm) occasionally had visible conks on their stems, which is aligned with the slightly higher mean heart rot area in these species compared with the other Angiosperms in this study (Table 1).

### 4.2 Positive Spatial Autocorrelation in Heart Rot Incidence and Degree Across and Within Species

The second aim of this study was to quantify whether there are spatial hotspots of clustering in heart rot incidence in the UNDERC FDP. Heart rot fungi should not be dispersal limited in this system, which provides the null expectation that there should be no spatial structure in heart rot incidence in forests. Alternatively, heart rot fungi colonization is facilitated by wounds caused by disturbance (Mook 1969; Basham 1991). For example, tree falls or limb breakage in a locality can wound other trees and lead to an increased probability of heart rot fungi colonizing neighboring individuals and, thereby, causing hotspots of heart rot. The heart rot‐bole breakage‐wound cycle proposed by Hennon (1995) posits that tree falls are, themselves, driven by heart rot. This would generate a spatial hotspot in heart rot incidence and it would also generate a hotspot that persists through time. As most heart rot fungi are not species‐specific (Gilbertson 1980; van der Wal et al. 2015), it is possible that there should be spatial hotspots of heart rot across species. We tested this expectation and found that, indeed, there are spatial hotspots in the incidence and percentage of heart rot across species in this study (Figure 2). These hotspots were roughly estimated to be the spatial grain of most canopy disturbances (i.e., tree falls). However, we note that the hotspots may actually be larger than this and this could not be detected given our study design, which relied on 20 × 20 m subplots that do not directly border one another. Thus, there is clear evidence that the incidence and percentage of heart rot across dominant canopy tree species in this forest has a spatial signature as would be expected by the heart rot‐bole breakage‐wound cycle.

Next, we investigated whether there are hotspots of heart rot within individual species. We limited this part of the study to the four species (*Ab. balsamea, Ac. saccharum, P. grandidentata*, and *P. tremuloides* ) as these species had enough individuals for robust inferences. There was local positive spatial autocorrelation in the incidence and percentage of heart rot in *Ab. balsamea* and in the percentage of heart rot in *P. tremuloides* (Figures 3 and 4). There was no spatial autocorrelation in heart rot within *P. grandidentata* and positive autocorrelation in *Ac. saccharum*, the most common tree in the study, only at moderate scales (Figures 3 and 4). Thus, the spatial autocorrelation in the incidence and percentage of heart rot across species was not reflected in all species, nor was this pattern driven by autocorrelation in the most common species. Rather, the general spatial autocorrelation in heart rot may be inferred to be driven by an underlying spatial variable independent of the distributions of individual species. Among the potential spatial mechanisms underlying these patterns are the heart rot‐bole breakage‐wound cycle mechanism and/or an underlying soil moisture gradient (Basham 1967; Basham 1973; Basham 1991; Wei et al. 2022). The final portion of this study aimed to test expectations arising from these mechanisms.

### 4.3 Heart Rot Along a Soil Moisture Gradient

The heart rot‐bole breakage‐wound cycle, as noted above, is expected to result in spatial hotspots of heart rot within and across species. An alternative, and not necessarily mutually exclusive, explanation for spatial hotspots could be an underlying gradient in soil moisture. Specifically, previous work has indicated that heart rot is more common in drier‐higher elevations in temperate forests presumably due to anoxic soil conditions limiting fungal growth (Basham 1991). To test this expectation, we quantified the correlation between soil moisture and the amount of heart rot within species using generalized linear models. We found no correlation between soil moisture and the incidence or percentage of heart rot within any of the species. Thus, the spatial autocorrelation in heart rot was not linked to soil moisture. We do acknowledge that studies spanning broader soil moisture gradients or soil moisture gradients in other forest types may find a relationship with heart rot, but there is no evidence for such a relationship in the present forest. The results are, however, consistent with the expected spatial structure of heart rot arising from the heart rot‐bole breakage‐wound cycle mechanism.

### 4.4 Conclusions

Heart rot fungi are an important but understudied biota in tree community ecology, with very little known about how heart rot incidence and magnitude vary across species and space in natural communities. The present work revealed that 71% of all large trees studied had some level of heart rot. However, the magnitude of heart rot was highly variable across individuals and was higher, on average, in the *Abies* species studied; there was no systematic variation in heart rot across species associated with their known life history differences. Previous work focusing on managed stands has hypothesized that heart rot should be nonrandomly distributed through space. In particular, heart rot may be more frequent or severe in drier soils, but our study found no evidence supporting this hypothesis. Conversely, we did find evidence that heart rot does spatially cluster across species in a manner consistent with what would be expected from heart rot‐bole breakage‐wound cycle mechanisms. Specifically, we expect that wounds caused by clusters of tree falls due to severe weather provide a pathway for fungal infection. This leads to an increase in heart rot incidence that, ultimately, will make these individuals prone to future bole failure, resulting in damage to neighboring individuals and additional cycles of heart rot fungal infection. These cycles, no doubt, play an important and underappreciated role in forest dynamics and health that require further study. For example, it is unclear how heart rot incidence and magnitude are related to tree growth, survival and reproduction, or seedling recruitment and density dependence. As tree community ecology continues to uncover the hidden roles of fungal interactions, we believe the widespread nature of heart rot will merit more attention.

## Author Contributions

**Hunter Gonzalez:** formal analysis (equal), investigation (equal), methodology (equal), writing – original draft (equal), writing – review and editing (equal). **Ally O'Neill:** investigation (supporting), methodology (supporting), writing – review and editing (supporting). **Michael Parent:** investigation (supporting), methodology (supporting), writing – review and editing (supporting). **Debit Datta:** data curation (supporting), investigation (supporting), methodology (supporting), writing – review and editing (supporting). **Nathan G. Swenson:** conceptualization (lead), data curation (supporting), formal analysis (supporting), funding acquisition (lead), investigation (supporting), methodology (supporting), project administration (equal), resources (lead), supervision (lead), visualization (supporting), writing – original draft (equal), writing – review and editing (equal).

## Conflicts of Interest

The authors declare no conflicts of interest.

## Data Availability

The data are available via Dryad at: http://datadryad.org/share/xsS6vGqSNjlAfyMpc92yGvixQHVeQoGlT4zWYSX39Zk.
