# Correction to “The Frequency, Magnitude, and Spatial Distribution of Heart Rot in Dominant Temperate Tree Species in a Forest Dynamics Plot”

**DOI:** 10.1002/ece3.72063

**Published:** 2025-08-27

Gonzalez, H., O'Neill, A., Parent, M., Datta, D. and Swenson, N.G. (2025), The Frequency, Magnitude, and Spatial Distribution of Heart Rot in Dominant Temperate Tree Species in a Forest Dynamics Plot. Ecol Evol, 15: e71329. https://doi.org/10.1002/ece3.71329

In the published article, a temporary URL was included in the Data Availability Statement. The data can now be accessed at the permanent URL: https://doi.org/10.5061/dryad.p2ngf1w2c.

We apologize for this error.

